# 3D fractal dimension analysis of CT imaging for microvascular invasion prediction in hepatocellular carcinoma

**DOI:** 10.1007/s00330-025-11878-6

**Published:** 2025-08-07

**Authors:** Feng Che, Qian Li, Wei Ren, Hehan Tang, Guli Zaina, Shan Yao, Ning Zhang, Shaocheng Zhu, Bin Song, Yi Wei

**Affiliations:** 1 Department of Radiology, West China Hospital, Sichuan University, Chengdu, China; 2 Department of CT Imaging Research Center, GE Healthcare China, Beijing, China; 3 Department of Radiology, Henan Provincial People’s Hospital, Zhengzhou, China; 4 Department of Radiology, Sanya Peoples Hospital, Sanya, China

**Keywords:** Hepatocellular carcinoma, Computed tomography, Fractal analysis, Microvascular invasion, Prognosis

## Abstract

**Objectives:**

This study aimed to assess the potential role of 3-dimensional (3D) fractal dimension (FD) derived from contrast-enhanced CT images in predicting microvascular invasion (MVI) in patients with hepatocellular carcinoma (HCC).

**Materials and methods:**

This retrospective study included 655 patients with surgically confirmed HCC from two medical centers (training set: 406 patients; internal test set: 170 patients; external test set: 79 patients). Box-counting algorithms were used to compute 3D FD values from portal venous phase images. Univariable and multivariable logistic regression analyses identified independent predictors. The model’s area under the curve (AUC) was calculated. Recurrence-free survival (RFS) and overall survival (OS) were evaluated using the Kaplan–Meier method.

**Results:**

Patients with MVI-positive HCC demonstrated significantly higher FD values compared to those with MVI-negative HCC (*p* < 0.01). The FD achieved AUCs of 0.786 (95% CI: 0.713–0.849) in the internal test set and 0.776 (95% CI: 0.669–0.874) in the external test set. A combined model incorporating alpha-fetoprotein, tumor size, tumor number, and FD showed superior diagnostic performance for MVI prediction compared to the clinical model, with AUCs of 0.795 (95% CI: 0.720–0.860) vs 0.752 (95% CI: 0.670–0.825) in the internal test set, and 0.826 (95% CI: 0.721–0.915) vs 0.739 (95% CI: 0.613–0.849) in the external test set. Patients stratified as high-risk MVI exhibited significantly worse RFS and OS outcomes compared to low-risk MVI patients (*p* < 0.05).

**Conclusion:**

The 3D FD values differed significantly between MVI-positive and MVI-negative HCC patients. Integrating FD into the clinical model enhanced MVI prediction accuracy and may help identify patients at high risk.

**Key Points:**

***Question*** *The predictive value of three-dimensional (3D) fractal dimension (FD) derived from contrast-enhanced CT images for identifying MVI-positive HCC remains unclear.*

***Findings*** *Quantitative indicators derived from fractal analysis were able to predict MVI. The developed model demonstrated improved performance when incorporating fractal dimension.*

***Clinical relevance*** *Fractal analysis based on contrast-enhanced CT is a feasible approach for evaluating MVI and provides additional clinical value for prognostic assessment. It may serve as a reference for preoperative MVI estimation and assist clinicians in executing more tailored therapies.*

**Graphical Abstract:**

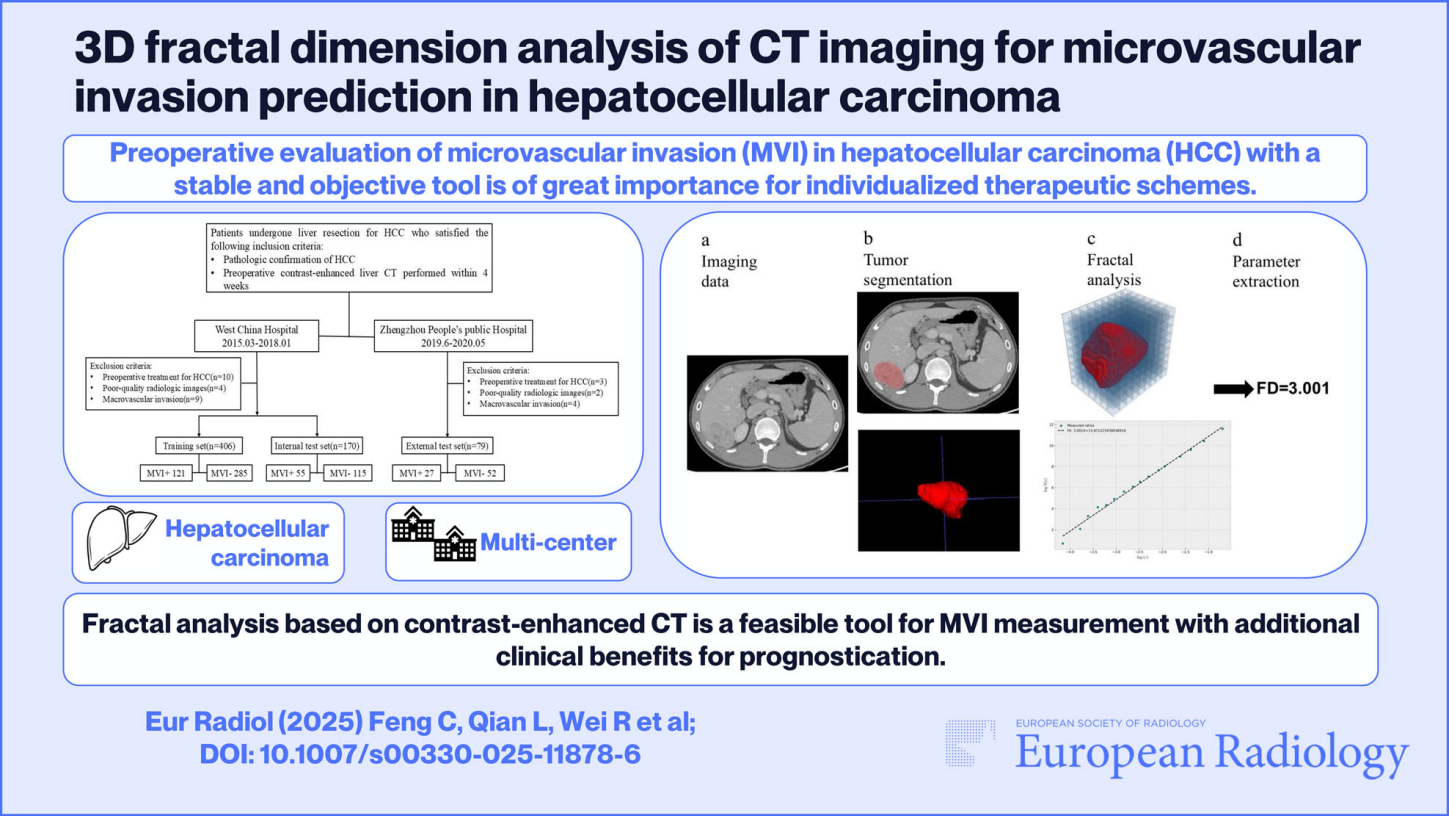

## Introduction

Hepatocellular carcinoma (HCC) is the most common primary liver cancer and the third leading cause of cancer-related death. Among numerous factors influencing long-term survival, microvascular invasion (MVI) is a well-established histopathological feature strongly associated with tumor recurrence and poor prognosis [1, 2]. Surgical resection in HCC patients with MVI often necessitates wider resection margins along with neoadjuvant or adjuvant therapies [3–5]. Moreover, the presence of MVI has also been one of the factors considered for liver transplantation due to its effect on the recurrence and overall survival of HCC patients receiving liver transplantation [6, 7]. Therefore, preoperative identification of MVI is crucial for optimizing clinical management and treatment planning.

A definitive diagnosis of MVI can only be obtained from a surgical specimen, limiting its applicability in guiding preoperative treatment decisions. Numerous studies have highlighted radiological features such as tumor margins, arterial rim enhancement, peritumoral hypointensity on hepatobiliary phase, and two-trait predictor of venous invasion as potential indicators of MVI [8–10]. Despite their high specificity, the subjective nature of evaluating these features has contributed to significant interobserver variability, raising concerns about their clinical applicability [11]. Other studies have also found that the clinical factors, including tumor size, tumor number and serum α-fetoprotein (AFP) levels, can be used for MVI prediction. Unfortunately, the clinical applicability of these serum markers for preoperative risk estimation of MVI remains to be determined. There is a critical need for stable and objective factors in MVI identification.

Fractal analysis is a mathematical technique that describes irregular or fragmented object shapes beyond traditional Euclidean geometry features. It offers a quantitative approach to assessing complex structural geometrics by calculating the fractal dimension (FD) [12, 13]. Branching structures like blood vessels have been shown to demonstrate fractal characteristics, rendering them highly appropriate for FD to assess tissue changes related to vascularization [13]. Previous applications of fractal analysis in radiological perfusion imaging have demonstrated its ability to characterize vascular patterns in rectal cancer patients and predict tumor grade in prostate cancer patients [14, 15]. Studies using pathological specimens have explored FD to quantify tumor infiltrative margin and predict prognosis [16, 17]. Although these studies have demonstrated their ability to evaluate tumor heterogeneity and microvascular complexity in cancers such as renal cell carcinoma and glioblastoma [18, 19], their potential role in assessing MVI in HCC remains unexplored. This mathematical approach provides a way to quantify the morphological characteristics induced by microvascular complexities and hemodynamic alterations, which holds great potential for MVI status evaluation in HCC patients.

Thus, the aim of this study was to evaluate the feasibility of fractal analysis using contrast-enhanced CT (CECT) for assessing MVI status in HCC patients and to determine its association with postoperative survival outcomes following curative surgical resection.

## Materials and methods

This study included retrospective data from two medical centers. The institutional review board approved this retrospective study, and informed consent was waived due to the study’s retrospective nature.

### Study population

**Fig. 1:**
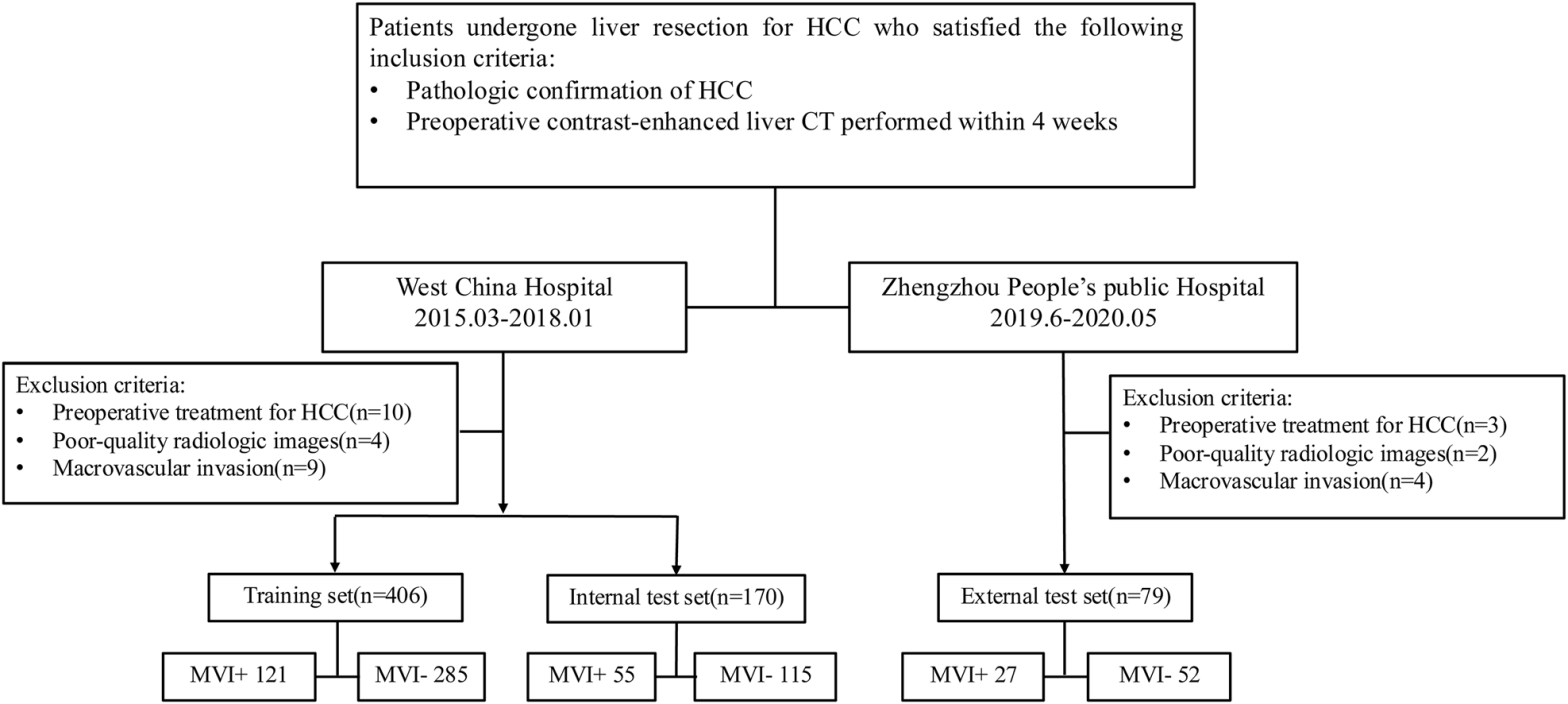
Flowchart of the study

Patients who underwent curative resection for HCC were identified retrospectively from two medical centers. At West China Hospital, patients from March 2015 to January 2018 were included and categorized into the training set and the internal test set in chronological order. Patients from Zhengzhou People’s Public Hospital between June 2019 and May 2020 constituted the external test set. Inclusion criteria were: (1) aged ≥ 18 years, (2) pathologically confirmed HCC, and (3) preoperative contrast-enhanced liver CT within 4 weeks. Exclusion criteria were: (1) prior treatment for HCC (e.g., radiofrequency ablation, transcatheter arterial chemoembolization), (2) poor-quality radiologic images, and (3) macroscopic vascular invasion. Figure 1 illustrates the detailed patient recruitment process.

Clinical data, including age, sex, hepatitis B virus infection, preoperative AFP, alanine aminotransferase (ALT), aspartate aminotransferase, gamma-glutamyl transferase, neutrophil-to-lymphocyte ratio (NLR), platelet count, prothrombin time, total bilirubin, and albumin (ALB), were collected from the electronic medical records system.

### Pathologic characteristics

MVI was microscopically observed as the presence of tumor cell nets in the portal vein, hepatic vein, or a large capsular vessel of the surrounding hepatic tissue lined with endothelium [20]. Histopathological characteristics (Edmondson-Steiner grade and liver fibrosis) were obtained from electronic health records. In patients with multiple HCCs, the largest tumor’s histopathologic information was utilized.

### CT protocols

Multiphasic CECT examinations were acquired for all patients. Imaging protocols are detailed in Supplementary Material S1 and Table S1.

### Image preprocessing and annotation

To standardize the CT images acquired from different scanners, image resampling and intensity normalization were performed. CT images were resampled to 1 × 1 × 1 mm^3^ voxel size by a linear interpolation method, and the Hounsfield units of CT images were normalized and quantized into 256 gray levels [21].

Tumor number and maximum tumor diameter were evaluated by two readers (reader 1 and reader 2, with 12 and 5 years of experience in abdominal imaging, respectively). They were informed that all enrolled patients had HCC but were unaware of the additional clinicopathological details. Three-dimensional lesion segmentation was performed by reader 1 using ITK-SNAP software (Version 3.8.0). Regions of interest were manually drawn on the portal venous phase (PVP) images, covering the whole tumor. In cases of multiple tumors, the largest tumor was selected as the main object. We randomly chose 100 patients to evaluate inter-reader and intra-reader reproducibility. Reader 1 repeated tumor segmentation twice in a 1-week period, and reader 2 independently performed the segmentation.

### Fractal analysis

**Fig. 2:**
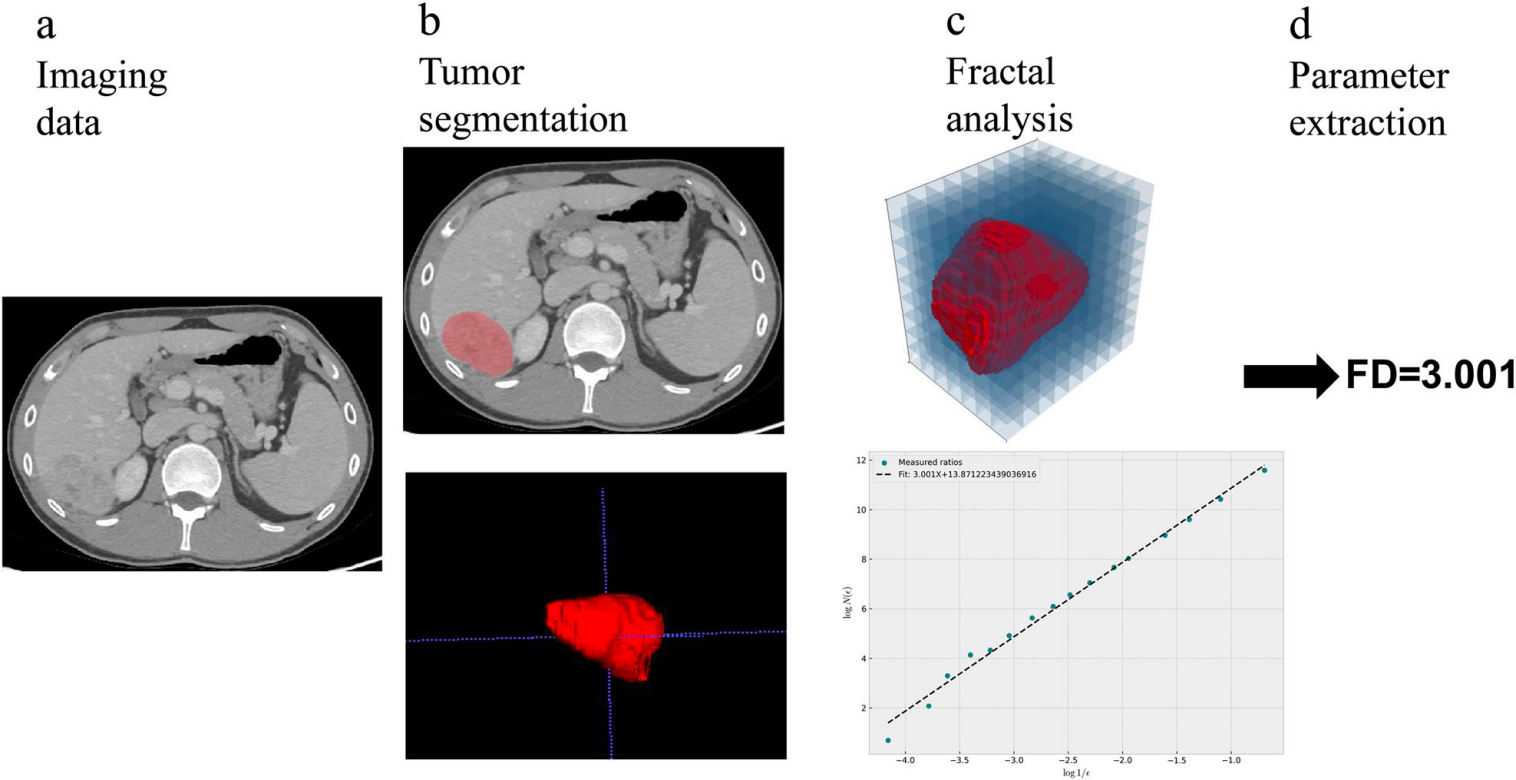
The main procedure of image data processing. **a** Imaging data acquisition. **b** Tumor segmentation. **c** Fractal analysis of the 3D tumor using the box counting method. **d** The estimated fractal dimention value of the tumor

Fractal analysis was conducted using Python 3.10 to comprehensively assess tumor morphology. This analysis involved the generation of graphical representations of tumors and the computation of their fractal dimensions using the box-counting method. Furthermore, sophisticated curve-fitting techniques were applied to derive precise fractal dimension curves, providing nuanced insights into tumor complexity. These findings were conveyed through detailed tumor box plots, offering spatially explicit representations of fractal dimension distributions. Notably, particular attention was given to determining the maximum fractal dimension of the primary lesion, serving as a crucial metric for comprehensive tumor assessment. Details of FD calculations are available in Supplementary Material S2. The main procedure of image data processing is shown in Fig. 2.

### Follow-up surveillance

Patients were consistently followed after surgery with AFP and imaging examinations, including ultrasound, CT, or magnetic resonance imaging, every 3 months during the first 2 years and then every 6 months thereafter. Recurrence-free survival (RFS) was defined as the time from the date of surgery to the date of the first recurrence, metastasis, or last follow-up. Overall survival (OS) was measured as the interval from surgery to death from any cause or last follow-up.

### Statistical analyses

Differences in the distribution of categorical variables were assessed using the Pearson χ^2^ or Fisher’s exact test. Continuous variables were compared using Student’s *t*-test or the Mann–Whitney U test, as appropriate. Univariable and multivariable logistic regression analyses were used to identify the risk predictors for MVI status in the training set, and variables with *p* < 0.05 at univariable analysis were considered for the multivariate model. The optimal cut-off value for the risk score was evaluated based on the best Youden’s index on the receiver operating characteristic (ROC) curve. The prediction performance was investigated with ROC curve analysis, and the areas under the curves (AUCs) were compared using the DeLong method. Calibration curves were plotted to assess the predicted values with actual values using the Hosmer–Lemeshow test. Decision curve analysis was used to evaluate the clinical utility. Interobserver variability was determined using the intraclass correlation coefficient (ICC) for continuous variables. Survival curves for RFS and OS according to pathological MVI and model-predicted MVI were generated using the Kaplan–Meier method and compared using the log-rank test.

Statistical analyses were implemented using R statistical software (version 4.1.0, http://www.r-project.org) and SPSS software (version 22.0, IBM). Two-sided *p*-values < 0.05 were considered significant.

## Results

### Patient characteristics

A total of 655 patients were included, with a median age of 53 years (range: 23–78 years). Baseline characteristics of patients in the training, internal test, and external test sets are detailed in Table 1. Most patients had hepatitis B virus infection (88% in the training set, 84% in the internal test set, and 86% in the external test set). Histologic MVI was present in 121 patients (29.8%) in the training set, 55 patients (32.4%) in the internal test set, and 27 patients (34.2%) in the external test set. Detailed patient characteristics according to MVI status in the training set are provided in Table S2.

**Table 1:** Baseline characteristics of patients

Variable	Training set (*n* = 406)	Internal test set (*n* = 170)	External test set (*n* = 79)
Age (years)*	51* (23–78)	52* (21–77)	56* (35–77)
Sex			
Male	356 (87.7)	137 (80.6)	67 (84.8)
HBV infection	359 (88.4)	142 (83.5)	68 (86.1)
Liver cirrhosis	268 (66.0)	88 (51.8)	43 (54.4)
AFP (ng/mL)			
> 400	153 (37.7)	67 (39.4)	19 (24.0)
ALT (IU/L)			
> 50	129 (31.8)	48 (28.2)	23 (29.1)
AST (IU/L)			
> 40	171 (42.1)	77 (45.2)	31 (39.2)
GGT (IU/L)			
> 45	272 (67.0)	119 (70.0)	54 (68.4)
NLR			
> 1.52	331 (81.5)	131 (77.1)	60 (75.9)
PLT (× 10^9^/L)			
> 100	289 (71.2)	121 (71.2)	63 (79.7)
PT (s)			
< 9.6 or > 12.8	105 (25.9)	35 (20.6)	15 (19.0)
TB (µmol/L)			
> 20.4	78 (19.2)	25 (14.7)	15 (19.0)
ALB (g/L)			
> 40	265 (65.3)	124 (72.9)	43 (54.4)
Child-Pugh			
A	372 (91.6)	158 (92.3)	73 (92.4)
Maximum tumor diameter (cm)			
> 5	221 (54.4)	88 (51.8)	27 (34.2)
Tumor number			
Solitary	363 (89.4)	153 (90.0)	63 (79.7)
Edmondson-Steiner grade			
I-II	230 (56.7)	102 (60.0)	47 (59.5)
MVI	121 (29.8)	55 (32.4)	27 (34.2)

Unless otherwise indicated, data are numbers of patients, and data in parentheses are percentages

*HBV* hepatitis B virus, *AFP* alpha-fetoprotein, *ALT* alanine aminotransferase, *AST* aspartate aminotransferase, *GGT* gamma-glutamyl transferase, *NLR* neutrophil-to-lymphocyte ratio, *PLT* platelet count, *PT* prothrombin time, *TB* total bilirubin, *ALB* serum albumin

* Data are medians, with interquartile ranges in parentheses

### Inter-reader reliability

Inter-reader reliability was excellent, with an ICC value of 0.98 (95% CI: 0.98–0.99) for FD. No statistical significance was found in the FD values for both MVI-positive and MVI-negative HCC between the two readers (Table S3). The interobserver agreement for tumor size and number was also excellent, with ICC values of 0.97 (95% CI: 0.95–0.98) and 0.96 (95% CI: 0.94–0.97), respectively.

### Predictive role of 3D FD with MVI status

**Fig. 3:**
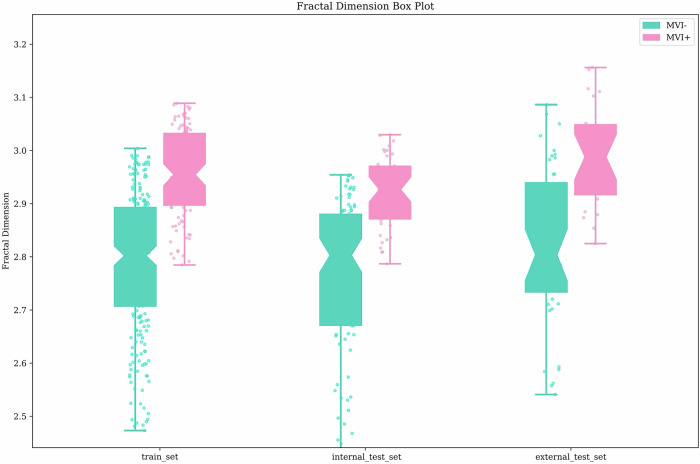
Comparison between fractal dimension (FD). Patients with MVI-positive HCC exhibit a notably higher FD value compared to those with MVI-negative cases

**Fig. 4:**
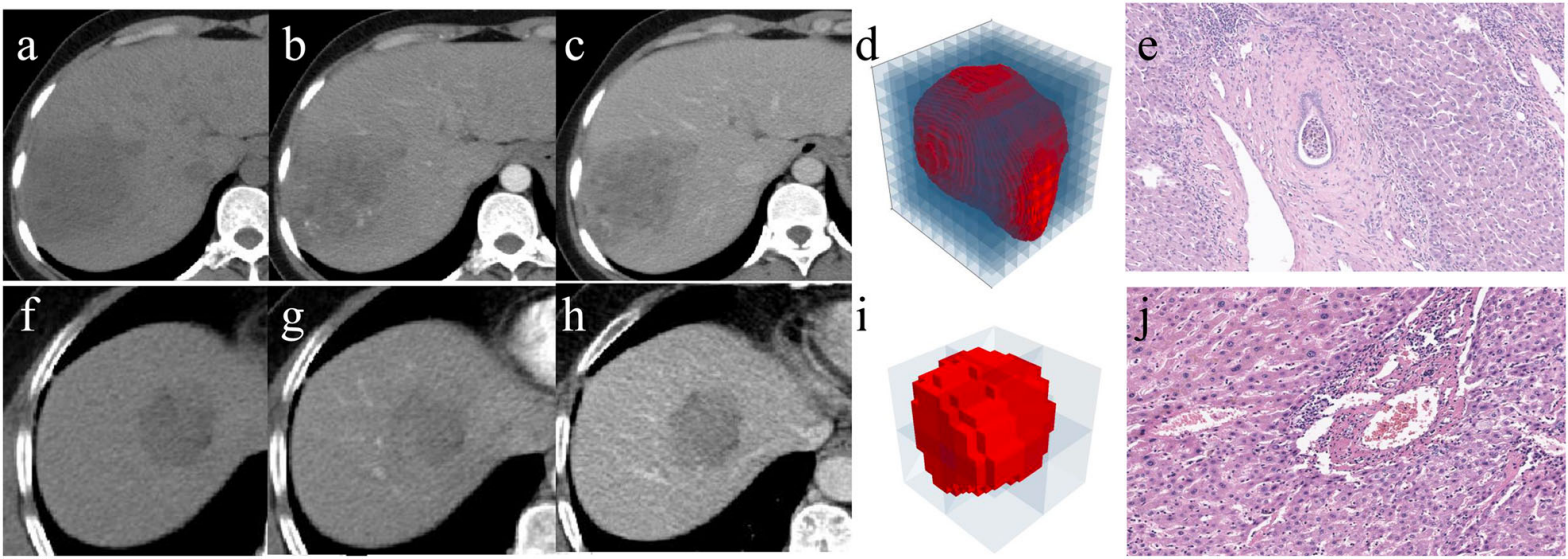
Case1: **a**–**e** Images in a 64-year-old man with MVI-positive HCC. **a**–**c** Axial CECT images acquired during the plain phase, AP, and PVP demonstrate a tumor measuring 8.3 cm in size. **d** Fractal analysis of the 3D tumor was performed using the box-counting method, yielding a FD of 2.96. **e** Histopathological image illustrates the presence of MVI (HE ×100). Case2: **f**–**j** Images in a 53-year-old man with MVI-negative HCC. **f**–**h** Axial CECT images obtained during the plain phase, AP, and PVP reveal a tumor measuring 4.0 cm in size. **i** Fractal analysis of the 3D tumor conducted via the box-counting method yielded a FD of 2.60. **j** Histopathological image demonstrates the absence of MVI (HE ×100). AP, arterial phase; PVP, portal venous phase; HE, hematoxylin-eosin staining

Significant differences in FD were detected between MVI-positive and MVI-negative HCC patients across all cohorts (Fig. 3). FD values were higher in MVI-positive patients compared to MVI-negative patients across all cohorts: training set (mean ± SD: 2.95 ± 0.10 vs 2.78 ± 0.19; *p* < 0.001), internal test set (mean ± SD: 2.92 ± 0.09 vs 2.72 ± 0.67; *p* < 0.001), and external test set (mean ± SD: 2.99 ± 0.15 vs 2.81 ± 0.22; *p* < 0.001) (Fig. 4). The AUCs for FD were 0.792 (95% CI: 0.747–0.838) in the training set, 0.786 (95% CI: 0.713–0.849) in the internal test set, and 0.776 (95% CI: 0.669–0.874) in the external test set.

### Model construction and comparison

**Fig. 5:**
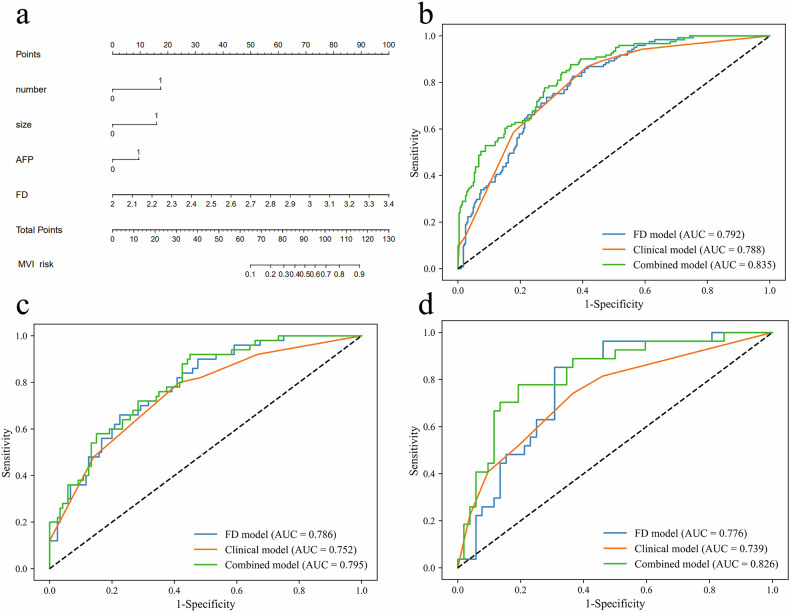
The developed combined nomogram (**a**). Predictive performance of the FD, clinical model, and combined model for MVI with receiver operating characteristic curve analysis in training set (**b**), internal test set (**c**), and external test set (**d**)

Univariable analysis identified AFP, ALT, NLR, ALB, tumor number, maximum tumor diameter, Edmondson-Steiner grade, and FD as variables associated with MVI status (all *p* < 0.05). After multivariable analysis, AFP (OR = 2.06, 95% CI: 1.21–3.49; *p* = 0.008), tumor number (OR = 3.89, 95% CI: 1.65–9.19; *p* = 0.002), maximum tumor diameter (OR = 3.99, 95% CI: 2.10–7.58; *p* < 0.001), and FD (OR = 62.39, 95% CI: 9.69–401.79; *p* < 0.001) were shown to be independent predictors of MVI (Table 2). The combined model was constructed integrating FD with clinical variables (AFP, tumor number, and maximum tumor diameter) and demonstrated good accuracy in estimating the risk of MVI. The multicollinearity analysis indicated that none of the variables exhibited excessively high collinearity, with VIF values ranging from 1.003 to 1.352 (Table S4). The AUCs for the combined model and the clinical model were 0.835 (95% CI: 0.793–0.874) and 0.788 (95% CI: 0.742–0.835) in the training set, 0.795 (95% CI: 0.720–0.860) and 0.752 (95% CI: 0.670–0.825) in the internal test set, and 0.826 (95% CI: 0.721–0.915) and 0.739 (95% CI: 0.613–0.849) in the external test set. The DeLong test demonstrated a significant improvement in diagnostic performance of the combined model over the clinical model (all *p* < 0.01) (Fig. 5, Table 3).

**Table 2:** Logistic regression analysis of variables for their association with microvascular invasion in patients in the training set

Variable	Univariate analysis		Multivariate analysis	
	OR (95% CI)	*p*-value	OR (95% CI)	*p*-value
Age (≤ 50 vs > 50 years)	0.53 (0.34–1.82)	0.812		
Sex (male vs female)	0.80 (0.43–1.50)	0.693		
HBV infection (absent vs present)	1.45 (0.71–2.95)	0.316		
Liver cirrhosis (absent vs present)	0.22 (0.01–0.03)	0.423		
AFP (≤ 400 vs > 400 ng/mL)	3.70 (2.37–5.78)	< 0.001	2.19 (1.30–3.70)	0.003
ALT (≤ 50 vs > 50 IU/L)	1.74 (1.12–2.72)	0.010	1.40 (0.82–2.41)	0.221
AST (≤ 40 vs > 40 IU/L)	1.34 (0.96–3.01)	0.391		
GGT (≤ 45 vs > 45 IU/L)	1.01 (1.32–3.64)	0.547		
NLR (≤ 1.52 vs > 1.52)	2.08 (1.11–3.89)	0.021	1.52 (0.72–3.18)	0.272
PLT (≤ 100 vs > 100 × 10^9^/L)	1.05 (0.66–1.69)	0.844		
PT (< 9.6 or > 12.8 vs 9.6–12.8 s)	0.57 (0.50–2.04)	0.692		
TB (≤ 20.4 vs > 20.4 µmol/L)	1.23 (0.72–2.08)	0.453		
ALB (≤ 40 vs > 40 g/L)	0.57 (0.37–0.89)	0.012	1.04 (0.61–1.80)	0.876
Child-Pugh (A vs B)	1.67 (0.62–7.32)	0.246		
Maximum tumor diameter (≤ 5 vs > 5 cm)	8.78 (4.99–15.45)	< 0.001	4.16 (2.19–7.89)	< 0.001
Tumor number (solitary vs multiple)	2.51 (1.32–4.77)	0.005	3.87 (1.63–9.23)	0.002
FD	146.99 (22.11–977.34)	< 0.001	62.21 (9.50–389.99)	< 0.001

Data in parentheses are 95% CIs

*HBV* hepatitis B virus, *AFP* alpha-fetoprotein, *ALT* alanine aminotransferase, *AST* aspartate aminotransferase, *GGT* gamma-glutamyl transferase, *NLR* neutrophil-to-lymphocyte ratio, *PLT* platelet count, *PT* prothrombin time, *TB* total bilirubin, *ALB* serum albumin, *OR* odds ratio

**Table 3:** Performance of the prediction model with and without FD

Data set	FD	Clinical model	Combined model	*p*-value
Training set				
SE (%)	81.6	86.8	87.6	0.881^a^
SPE (%)	66.2	58.6	65.9	0.032^b^
ACC (%)	73.0	67.0	75.9	
AUC (95% CI)	0.792 (0.747–0.838)	0.788 (0.742–0.835)	0.835 (0.793–0.874)	< 0.001^c^
Internal test set				
SE (%)	69.0	80.0	78.0	0.434^a^
SPE (%)	77.5	58.3	66.0	0.758^b^
ACC (%)	74.1	64.7	75.9	
AUC (95% CI)	0.786 (0.713–0.849)	0.752 (0.670–0.825)	0.795 (0.720–0.860)	0.002^c^
External test set				
SE (%)	85.2	74.1	77.8	0.647^a^
SPE (%)	69.2	63.5	80.8	0.362^b^
ACC (%)	74.7	67.1	79.7	
AUC (95% CI)	0.776 (0.669–0.874)	0.739 (0.613–0.849)	0.826 (0.721–0.915)	0.004^c^

*SEN* sensitivity, *SPE* specificity, *ACC* accuracy, *AUC* area under the receiver operating characteristic curve

^a^ FD vs clinical model

^b^ FD vs combined model

^c^ Clinical model vs combined model

The calibration curves of the models demonstrated good agreement between predicted and observed MVI across three cohorts (Fig. S1). Decision curve analysis revealed superior net benefit for the combined model compared to the clinical model across the range of reasonable threshold probabilities (Fig. S2).

### Prognostic analysis

**Fig. 6:**
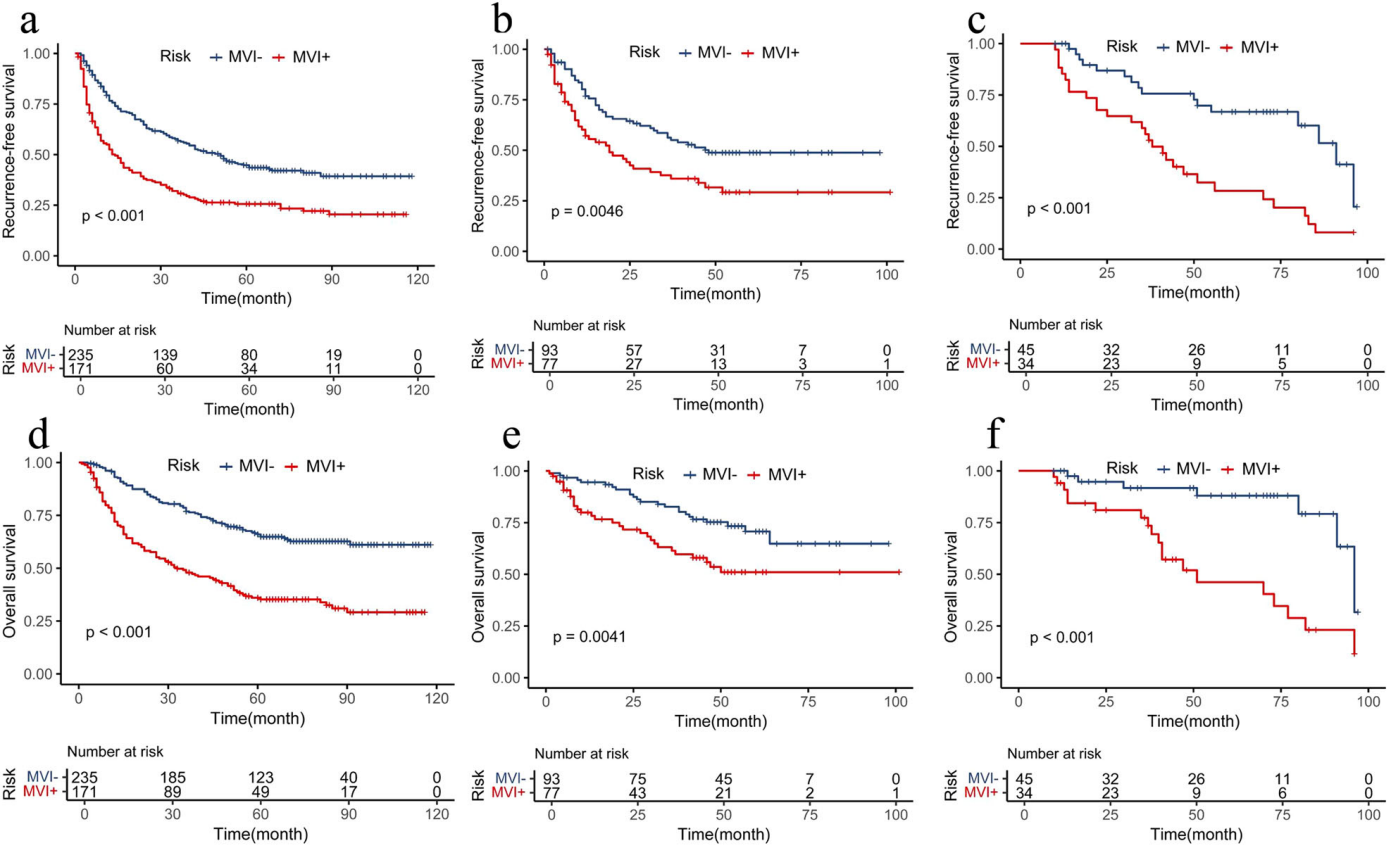
Kaplan–Meier curves of recurrence-free survival in the training (**a**), internal test (**b**), and external test set (**c**), and overall survival in the training (**d**), internal test (**e**), and external test set (**f**) according to the combined model

The histologic MVI-positive patients had poorer RFS and OS compared with MVI-negative patients in all cohorts (all *p* < 0.05) (Fig. S3). Based on the predicted scores from the combined model (best cut-off value −0.76, 95% CI: 0.74–0.79), patients were stratified into high-risk and low-risk groups. Patients predicted to have high-risk MVI showed significantly worse prognosis in the training set, with median RFS 28 months [IQR, 4–45] and median OS 40 months [IQR, 11–63] compared to low-risk MVI (median RFS 43 months [IQR, 12–68] and median OS 58 months [IQR, 36–81]) (all *p* < 0.01, Fig. 6). Similar results were observed in the internal test set (RFS: *p* = 0.0046; OS: *p* = 0.0041) and external test set (*p* < 0.001 for both) (Fig. 6).

## Discussion

In this study, we first evaluated the predictive value of 3D fractal analysis from large-scale CECT images for preoperative MVI status evaluation in 655 HCC patients. Integrating FD with clinical factors, our combined model demonstrated robust predictive performance for MVI status, which was validated in an independent cohort. Based on the combined model, patients were stratified into two prognostically distinct risk categories. Those identified as high-risk MVI exhibited poorer survival outcomes and were more likely to benefit from more aggressive treatment approaches.

The preoperative prediction of MVI remains challenging, and there is currently a lack of highly reliable factors to predict MVI. In this study, fractal analysis was used for MVI status evaluation in HCC patients, and higher FD values were observed in MVI-positive groups compared to MVI-negative groups. Previous studies have applied fractal analysis for tumor differentiation and biological characteristics visualization based on its ability to describe irregular structures from different dimensions and quantify tumor complexity [22–24]. Michallek et al found that FD can predict prostate cancer grade based on multiparametric MRI and adds independent information into PI-RADS assessment [25, 26]. The correlation between the fractal parameter and hidden dimensions of MVI from CT images may be attributed to several factors. First, tumor emboli or clusters of cancer cells in the branches of hepatic vessels can induce hemodynamic alterations in the adjacent hepatic tissue, resulting in irregular tumor margins and increased texture complexity. This morphological characteristic can be quantified by FD values, which reflect conditions localized at the microvascular level. Al-Kadi et al also reported that fractal analysis could offer further insights into the aggressiveness of tumors [27]. Second, branching structures like blood vessels demonstrate fractal characteristics, making it highly appropriate for FD to assess tissue changes related to vascularization [13]. Many previous studies have reported the utility of fractal analysis in evaluating neovascularization changes and quantifying tumor vascularity [28–30]. It is precisely due to this advantage that microvascular changes in MVI-positive HCC can be effectively captured and quantified using FD. However, further exploration is needed to fully understand these potential mechanisms.

In the current study, FD was derived from PVP images, which were reported to exhibit better reproducibility compared to AP [31]. MVI is a pathological feature mainly seen in the small branches of the portal vein in the tumor capsule and/or noncapsular fibrous septa [32, 33]. In the study by Banerjee et al, a radiogenomic marker based exclusively on PVP images was demonstrated to accurately predict MVI [34]. Another study also reported that PVP radiomics signature performed better than AP, DP, or any kind of combined radiomics signatures [35]. This may further explain the satisfactory performance of the fractal parameter for MVI prediction from PVP. Importantly, our study advanced from prior 2D analyses by applying 3D fractal analysis, comprehensively capturing tumor structural characteristics from different dimensions. By employing box-counting methods across the entire tumor volume, we accurately measured the non-integer dimensional features critical for understanding tumor heterogeneity characteristics hidden behind the visible imaging. Although fractal analysis appears promising as a quantitative marker, this technique necessitates additional image processing, including tumor segmentation, which currently constitutes up to 90% of the analysis time (30 min per patient, compared to 3 min for box-counting). This is time-consuming and labor-intensive for clinical applications. With recent advances in artificial intelligence algorithms, automated tumor segmentation may help improve efficiency and reproducibility [36, 37]. Moreover, while FD provides a quantitative assessment of tumor morphological complexity, whether other histopathological factors, such as tumor differentiation and peritumoral inflammation, could also contribute to the observed variations remains unknown. Future prospective studies are warranted to confirm our findings and mitigate potential confounding factors.

Clinical factors, including serum AFP level, tumor size, and number,r have previously been shown to be associated with MVI status and were integrated into our clinical model. These preoperative factors have been confirmed to be closely related to tumor biological behavior and prognosis in HCC patients [38, 39]. The incorporation of FD along with these factors significantly enhanced the predictive performance in both the internal and external test sets, underscoring the utility of FD analysis. Various semantic imaging features have been described for the prediction of MVI, but their diagnostic utility varies. The subjectivity of semantic imaging feature assessment is one of the important factors affecting diagnostic efficacy and reproducibility. In this study, a quantitative approach was used, which provides objective metrics for assessing tumor MVI status. Additionally, previous studies have demonstrated the potential of CT radiomics for MVI prediction while requiring manual selection from a vast array of human-defined features [35, 38, 40, 41]. This process can be subjective and carries the risk of overfitting. Furthermore, the radiomics features extracted using different software and packages may vary, leading to instability in the features, which compromises their reproducibility and diminishes the applicability of the results in clinical practice. Other quantitative image markers based on CT, such as iodine concentration, atomic number, and perfusion, have also been reported to be related to MVI [42–47]. However, the power of these studies has been limited by both a small sample size (e.g., 36–104 patients) and a lack of external validation. Our study applied this user-friendly tool for MVI assessment in a large cohort with external validation and yielded encouraging results. Unlike traditional radiomic features that rely on fixed-scale intensity and texture analysis, fractal features capture the complexity and self-similarity of tumor morphology across scales. FD provides a scale-invariant and potentially more biologically relevant descriptor of tumor heterogeneity. Furthermore, our findings demonstrate that patients predicted to have positive MVI exhibit poorer RFS and OS outcomes compared to those with negative MVI. This highlights the potential value of preoperative risk stratification for prognostic assessment and for refining personalized treatment strategies.

Some limitations of our study should be acknowledged. First, despite efforts to mitigate bias through chronological division of training and internal test sets, the retrospective nature of our study introduces inherent limitations. Further prospective and multicenter studies should be conducted. Second, perfusion imaging has been used as a proxy for detecting and assessing microvascular alterations [14]. Changes in FD values across different perfusion phases may be more closely related to hemodynamic variations caused by MVI status. In our study, 3D fractal features were analyzed exclusively on the PVP. Future research may extend fractal analysis to additional contrast-enhanced phases or perfusion imaging to further enhance the evaluation of MVI. Third, the utilization of different CT scanners from the two centers may influence the fractal parameter. One advantage of FD calculation is its relative resilience to image noise effects. Al-Kadi examined the impact of noise on CT images regarding FD, noting that FD and wavelet packet transform analyses were the least affected by noise [48].

In conclusion, the quantitative indicators derived from fractal analysis provide promising diagnostic markers and valuable information regarding the microvascular structural characteristics of tumors. Furthermore, when combined with clinical models, FD can enhance the predictive performance of MVI. This method of non-invasive and precise measurement of MVI can further provide clinical benefits for prognostic prediction and may serve as a reference for guiding individualized treatment in the management of HCC patients.

## Supplementary information

ELECTRONIC SUPPLEMENTARY MATERIAL

## Abbreviations

AFP: Alpha-fetoprotein
AUC: Area under the receiver operating characteristic curve
CECT: Contrast-enhanced CT
FD: Fractal dimension
HBV: Hepatitis B virus
HCC: Hepatocellular carcinoma
ICC: Intraclass correlation coefficient
IQR: Interquartile range
MVI: Microvascular invasion
OR: Odds ratio
OS: Overall survival
RFS: Recurrence-free survival
