# Supplementary material for: 3D fractal dimension analysis of CT imaging for microvascular invasion prediction in hepatocellular carcinoma
Source: Eur Radiol. 2025 Aug 7;36(2):1351–62. doi: 10.1007/s00330-025-11878-6 (PMC12953277; doi:10.1007/s00330-025-11878-6)

3D fractal dimension analysis of CT imaging for microvascular invasion prediction in hepatocellular carcinoma

ELECTRONIC SUPPLEMENTARY MATERIAL

S1. CT Imaging Protocol

CECT examinations were performed using multidetector CT scanners (Revolution, GE Healthcare, Milwaukee, USA; SOMATOM Definition, Siemens Healthcare, Erlangen, Germany). After acquiring nonenhanced images, a bolus of nonionic contrast agent (1.5-2.0mL/kg; omnipaque 350 mg/mL, GE Healthcare Chicago, IL) was injected at 3.0 mL/s. Arterial phase images (at trigger) and portal venous phase images (25-30 seconds post-trigger) were obtained with a trigger threshold of the aorta reaching 100 HU. CT scanners parameters were listed as follow.

S2. Fractal analysis by box counting method

The MONOAI (Medical Open Network for AI) framework was utilized for the segmented tumor volumes preprocessing and analysis. The preprocessing began with loading the segmented tumor volumes using the LoadImaged function, followed by standardizing voxel dimensions with Spacingd to ensure isotropic resolution. Next, the region of interest was cropped using CropForegroundd to remove background noise. Finally, the processed images were converted into NumPy arrays for subsequent fractal computation. These sequential steps facilitated the efficient and reproducible extraction of the relevant quantitative features from the imaging data.

In this study, the widely used box-counting method was employed to estimate the fractal dimension (FD) of tumor regions. To improve robustness and reduce bias due to box placement, multiple grid offsets were introduced during the calculation. The number of boxe $B(s)$ required to cover the segmented region at a given scale s follows the relationship:

$$B(s) = C * s^{-F}$$

where $B(s)$ represents the number of boxes of size s, C is a proportionality constant, and F is the FD that reflects the structural complexity of the tumor. To determine the fractal dimension, a log-log plot of $B(s)$ against sss was constructed, and the slope of the fitted regression line was used to estimate F:

$$B(s) = -\frac{dlogB(s)}{dlog\ s}$$

Table S1 CT scanners parameters

Parameters	GE Healthcare	Siemens Healthcare
kV	90-120	90-120
mA	200-210	200-210
Pitch	0.992	1.0
Rotation time	0.5 s/rot	0.5 s/rot
Section thickness	0.625 mm	0.5 mm

Table S2 Patient characteristics in the training set according to the MVI status

Variables	MVI+ (n=121)	MVI- (n=285)	*P* Value
Age(y) *	47 (23-73)	52 (23-78)	0.419
Sex male	104 (86.0)	252 (88.4)	0.511
HBV infection	110 (90.9)	249 (87.4)	0.297
Liver cirrhosis	92 (76.0)	176 (61.8)	0.375
AFP level (ng/ml)			
>400	72 (59.5)	81 (28.4)	< 0.001
ALT (U/L)			
>50	49 (40.5)	80 (28.1)	0.005
AST (U/L)			
>40	52 (43.0)	119 (41.8)	0.284
GGT (U/L)			
>45	86 (71.1)	186 (65.3)	0.453
NLR			
>1.52	107 (88.4)	224 (78.6)	0.019
PLT ($\times 10^9$/L)			
>100	87 (71.9)	202 (70.9)	0.765
PT(s)			
< 9.6 or > 12.8	30 (24.8)	75 (26.3)	0.567
TB (μmol/L)			
>20.4	26 (21.5)	52 (18.2)	0.401
ALB (g/L)			
>40	68 (56.2)	197 (69.1)	0.016
Child-Pugh			
A	107 (88.4)	265 (93.0)	0.143
Maximum tumor diameter(cm)			
>5	104 (86.0)	114 (40.0)	< 0.001
Tumor number			
solitary	100 (82.6)	117 (41.0)	0.007
Edmondson–Steiner grade			
I-II	52 (43.0)	107 (37.5)	< 0.001
FD	2.95 ± 0.10	2.78 ± 0.19	< 0.001

Note. Unless otherwise indicated, data are numbers of patients, and data in parentheses are percentages. HBV, hepatitis B virus; AFP, alpha-fetoprotein; ALT, alanine aminotransferase; AST, aspartate aminotransferase; GGT, gamma-glutamyl transferase; NLR, neutrophil-to-lymphocyte ratio; PLT, platelet count; PT, prothrombin time; TB, total bilirubin; ALB, serum albumin; MVI, microvascular invasion; FD, fractal dimension.

* Data are medians, with interquartile ranges in parentheses.

Table S3 The FD between two readers in MVI-positive and MVI-negative groups.

	MVI+	MVI-
Reader1	2.96 ± 0.11	2.73 ± 0.18
Reader2	2.97 ± 0.10	2.75 ± 0.17
P value	0.780	0.893

Note. MVI, microvascular invasion; FD, fractal dimension.

Table S4 Collinearity analysis among variables of the combined model.

**Variables**	Collinearity Statistics	
	Tolerance	VIF
FD	0.740	1.352
Size	0.746	1.340
Number	0.997	1.003
AFP	0.902	1.108

Note: VIF, variance inflation factors; FD, fractal dimension.

Figure S1 The calibration curves of the FD, clinical model, and combined model in the training (**a**), internal test(**b**), and external test sets(**c**).

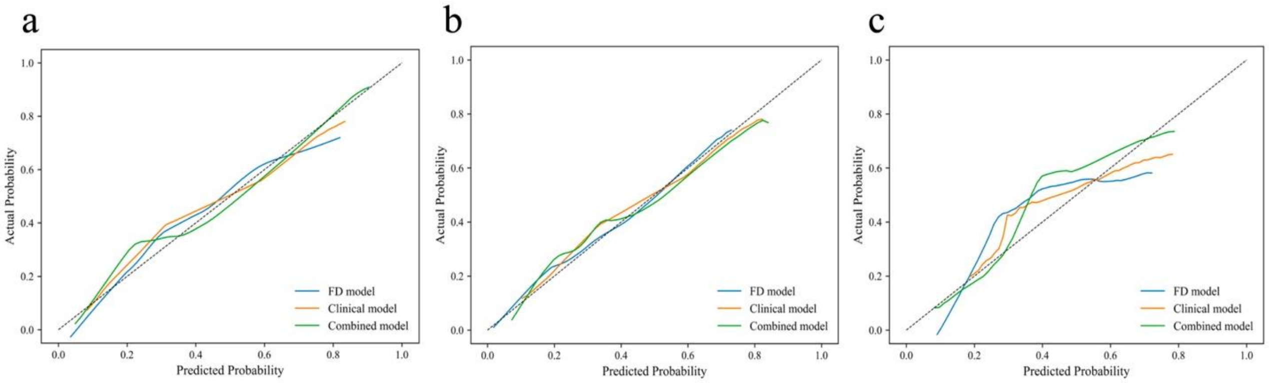

Note. FD, fractal dimension.

Figure S2 The decision curves of the FD, clinical model, and combined model in the training (**a**), internal test(**b**), and external test sets(**c**).

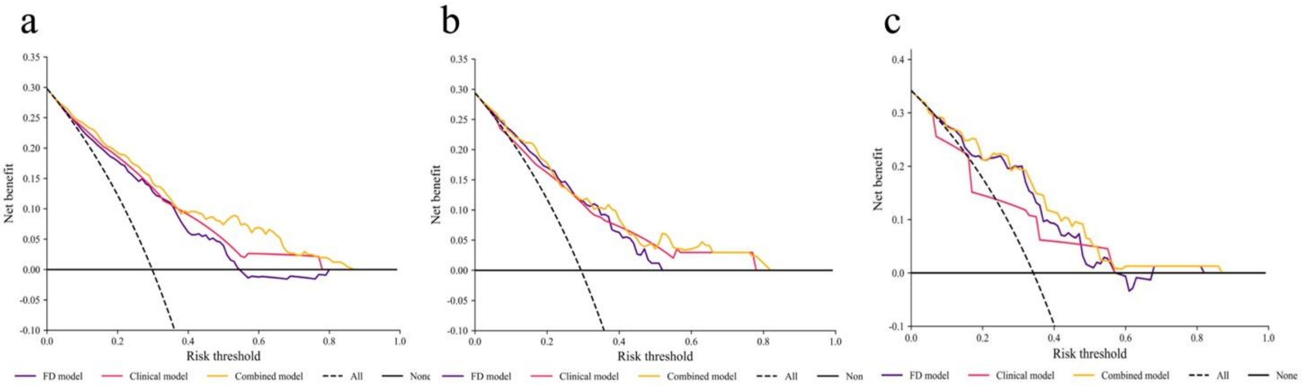

Note. FD, fractal dimension.

Figure S3 Kaplan-Meier curves of recurrence-free survival in the training**(a)**, internal test**(b)**, and external test set**(c)** and overall survival in the training**(d)**, internal test**(e)**, and external test set**(f)** according to pathological MVI.

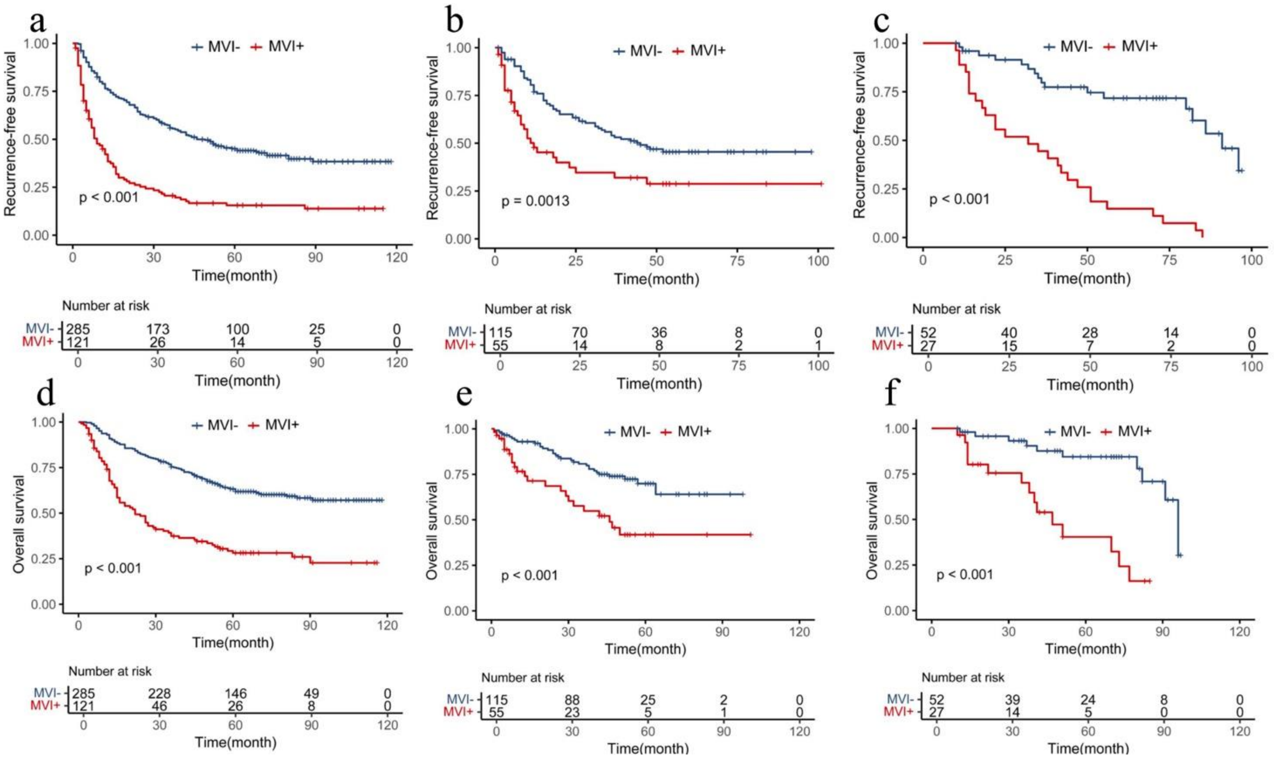

Supplement: Supplementary file 1 — ELECTRONIC SUPPLEMENTARY MATERIAL [file 330_2025_11878_MOESM1_ESM.pdf]
